# Green synthesis of copper oxide nanoparticles *ST@CuONPs* by *Streptomyces thinghirensis*: current treatment path against multidrug-resistant bacteria

**DOI:** 10.3389/fphar.2026.1904965

**Published:** 2026-10-15

**Authors:** Amal Loqman, Qassim Esmaeel, Stéphanie Sayen, Simon Remy, Jean-Hugues Renault, Younes Smani, Souad Loqman

**Affiliations:** 1 Laboratoire de Lutte Contre les Maladies Infectieuses, Faculté de Médecine et de Pharmacie, Université Cadi Ayyad, Marrakech, Morocco; 2 Université de Reims Champagne-Ardenne, INRAE, RIBP UMR 1488, Reims, France; 3 Molecular Chemistry Institute of Reims (ICMR), UMR CNRS 7312, University of Reims Champagne Ardenne, Reims, France; 4 Centro Andaluz de Biología del Desarrollo, Universidad Pablo de Olavide/CSIC/Junta de Andalucía, Seville, Spain; 5 Departamento de Biología Molecular e Ingeniería Bioquímica, Universidad Pablo de Olavide, Seville, Spain; 6 Laboratoire de Microbiologie, CHU Mohammed VI, Marrakech, Morocco

**Keywords:** agar disk-diffusion, biofilms, broth microdilution, copper oxide nanoparticles, multidrug-resistant bacteria, *Streptomyces thinghirensis*

## Abstract

**Background:**

Antibiotic resistance is currently a global concern, leading to serious complications in both clinical and non-clinical settings, and is particularly dreaded in developing countries. In the search for alternative treatments against bacterial infections, nanotechnology has opened up unexpected new horizons in the scientific world, thanks to the great variety of available nanomaterials that have potential antimicrobial activity. Some of the interest in this type of nanomaterials relies in their exceptional properties and usages in many fields. The investigation focused on the synthesis of a new copper oxide nanoparticles ST@CuONPs as a promising alternative to conventional antibiotics using *Streptomyces thinghirensis* (DSM 41919^T^) as a novel, bacteria-mediated synthesis pathway.

**Methods:**

The ST@CuONPs were confirmed and characterized by UV-Vis spectroscopy, Fourier transform infrared (FTIR) spectroscopy, particle-size analyzer, scanning electron microscopy (SEM), X-ray diffraction (XRD) and transmission electron microscopy (TEM). The antimicrobial and antibiofilm activities of ST@CuONPs were evaluated against Vancomycin-resistant *Enterococcus faecium* VRE ATCC 700221vanA, Methicillin-resistant *Staphylococcus aureus* NCTC 12493 and Ceftazidime-Resistant *Pseudomonas aeruginosa* ATCC BAA-2108, as well as Vancomycin-sensitive *Enterococcus faecalis* ATCC 29212, Methicillin-sensitive *Staphylococcus aureus* ATCC 29213 and Ceftazidime-sensitive *P. aeruginosa* ATCC 27853.

**Results and Discussion:**

TEM analysis revealed a spherical shape distribution of ST@CuONPs with an average size of ∼9.8 ± 0.27 nm. The ST@CuONPs demonstrated broad-spectrum antibacterial and antibiofilm activities to ward Multidrug-resistant bacteria. In addition, among the tested multi-drug resistant (MDR) pathogens, the highest antibacterial and antibiofilm efficacies were observed against the Gram-positive strain *E. faecium* VREATCC 700221vanA. The bacterial biofilm was reduced by 64% ± 0.58% at 1/4 × MIC, 83% ± 3% at 1/2 × MIC and 89% ± 1.15% at 1×MIC of ST@CuONP.

**Conclusion:**

The synthesis of ST@CuONPs using *S. thinghirensis* is an eco-friendly and economical method that avoids the use of toxic reagents. The resulting nanoparticles exhibit a potential to be explored as an alternative to conventional antibiotics.

## 1 Introduction

Multidrug-resistant (MDR) bacteria present a global challenge, posing a major risk to both human health and the environment. Biofilm formation has emerged as a key mechanism in the development of resistance. Bacterial species utilize bacterial cell-to-cell communication, known as quorum sensing, to regulate this physiological process (Rutherford and Bassler, 2012). With the rising bacterial resistance to conventional antibiotics, the search for new therapeutic strategies has regained prominence.

Nanoparticles, based on their utility as a potential antibacterial agent, have been widely used to avoid adverse effects and drug resistance induced by current treatments of bacterial infections (Yang et al., 2017; Zheng et al., 2017; Liu et al., 2019; Wang et al., 2019). The metal oxide nanoparticles can pass through living cells' membranes, destroy the active substances, and quickly kill bacteria (Kotrange et al., 2021). A vast array of techniques such as physical, chemical and biological could be applied for the production of nanosized particles (Nyabadza et al., 2023). However, chemical and physical techniques have their limitations in terms of cost instruments and contamination caused by toxic solvents introduced throughout the synthesis. Hence, the methods employed for the synthesis of nanoparticles need to be continuously refined.

Green production of nanoparticles also called biological methods have acquired increased attention due to their synthesis approach, which is simple, non-toxic, rapid, clean, inexpensive, and eco-friendly (Phull et al., 2016; Kumari R. et al., 2020). The first work has been published in 1980 by Beveridge and Murray which list the concept of using microorganisms *Bacillus subtilis* to synthesize nanosized gold (Beveridge and Murray, 1980). Until now, a wide range of biological communities as prospective nanoparticle synthesizers have been evaluated, including bacteria (Busi et al., 2014; Ramalingam et al., 2014; John et al., 2020), plants (Ahmed et al., 2016), yeast (Eugenio et al., 2016; Korbekandi et al., 2016), fungi (Ottoni et al., 2017; Molnár et al., 2018; Shahzad et al., 2019) and actinomycetes (Al-Dhabi et al., 2018; Hamed et al., 2020; Kumari S. et al., 2020; Loqman et al., 2022).

Actinomycetes have emerged as one of the ideal sources for medical and industrials products. Based on their secondary metabolites, they could be employed as a stabilizing agent for production of metals nano-scale (Bakhtiari-Sardari et al., 2020). A multitude research papers have documented the unique ability of *Streptomyces sp*. to synthesize diverse types of metallic nanoparticles, such as zinc, silver, manganese, gold and copper (Deshmukh and Kulkarni, 2011; Shivaji et al., 2014; El-Naggar et al., 2016; Hassan et al., 2018; Bukhari et al., 2021). Copper is a cost-effective material in comparison to other noble metals like gold, platinum, and silver. Generally, nanoscale dimensions of copper nanoparticles have been the key to many important applications such as anticancer (Gnanavel et al., 2017), antimicrobial (Almasi et al., 2018; Rajesh et al., 2018), antifungal (Priya et al., 2023), antioxidant activities (Rehana et al., 2017) and biofilm eradication (Elsayed et al., 2020).

The alarming rise of antimicrobial resistance (AMR) remains a profound threat to global public health, associated with millions of deaths annually (GBD, 2021 Antimicrobial Resistance Collaborators, 2024). In the natural environment, biofilms represent the preferred mode of bacterial life, significantly accentuating their virulence, survival in unfavorable conditions, and serving as critical hotspots for the accumulation and diffusion of antibiotic resistance genes (ARGs) (Beshiru et al., 2023; Michaelis and Grohmann, 2023; Naudin et al., 2024). These ARGs are easily exchanged between resistant and non-resistant bacterial strains, driving genetic transformation and accelerating their spread across human and animal communities (Sghaier et al., 2019; Avatsingh et al., 2023). To guide the development of novel therapeutics against such threats, the World Health Organization (WHO) updated its Bacterial Priority Pathogens List to classify these adaptable MDR strains by clinical urgency (WHO, 2024; Sati et al., 2025). Within this global framework, our target pathogens, resistant *Pseudomonas aeruginosa*, vancomycin-resistant *Enterococcus faecium* (VRE), and methicillin-resistant *Staphylococcus aureus* (MRSA) are identified as high priority threats (Sati et al., 2025). The continuous hazard of these high-burden organisms highlights the severe limitations of standard antibiotics, accentuating the need for innovative, non-conventional antimicrobial agents such as green synthesized CuONPs.

With the rapid progress of nanotechnology, the remarkable antibacterial effectiveness of copper nanoparticles against pathogens has been thoroughly studied (Amiri et al., 2017; Pugazhendhi et al., 2018; Padmavathi et al., 2020; Torres-Rosas et al., 2020). To date, there have been no works in the literature regarding the use of *Streptomyces thinghirensis* (*S. thinghirensis*) as a reducing agent for the production of CuONPs. In light of the above concerns, this work is focused on the fabricating *S. thinghirensis* Copper Oxide Nanoparticles (ST@CuONPs) through a green synthetic route by using *S. thinghirensis* as a stabilizing agent. Additionally, *E. faecalis* ATCC 29212 strain, *E. faecium* VREATCC 700221vanA strain, methicillin-susceptible *S. aureus* ATCC 29213 strain, methicillin-resistant *S. aureus* NCTC 12493 strain, susceptible *P. aeruginosa* ATCC 27853 and resistant *P. aeruginosa* ATCC BAA-2108 strain were employed to examine the antibacterial and antibiofilm effects of ST@CuONPs.

## 2 Materials and methods

### 2.1 Biosynthesis and analysis method of ST@CuONPs

The biosynthesis of ST@CuONPs was performed following a previously reported method, with minor modifications (Flores-Rábago et al., 2023). The protocol for collecting actinobacterial secondary metabolites of *S. thinghirensis* was adopted from Loqman et al. (Loqman et al., 2022). The collected supernatant was combined with a freshly prepared CuSO_4_·5H_2_O (10 mM) (Sigma-Aldrich, Allemagne) solution as a precursor in a 1:1 (v/v) ratio. The mixture was incubated in an orbital shaker at 30 °C in darkness for up to 120 h. A negative control, consisting of the supernatant without CuSO_4_·5H_2_O was included in the experiment. The production of ST@CuONPs was checked by a color shift from light blue to greenish brown using a UV-visible spectrophotometer (Secoman Uviline 9400, France). The ST@CuONPs synthesized *via* the green method was characterized using an X’Pert Pro Panalytical diffractometer (CuKα = 1.54060 Å) with an X’Celerator scintillation detector (30 mA, 40 kV) over a 10–80 range at a 2/min scanning rate. High-resolution transmission electron microscopy (HR-TEM) (FEI TecnaiG2, Allemagne) was employed to analyze morphology, elemental composition (EDX) and particle size. The crystalline structure and interplanar d-spacing were determined *via* SAED. Fourier transform infrared spectroscopy (FTIR) (IR-TF/Vertex 70V/Perkin-Elmer/Jasco-4600, Allemagne) identified functional groups, while the Zetasizer Nano ZS (Malvern, Angleterre) measured the zeta potential and average particle size of ST@CuONPs.

### 2.2 Evaluation of the effect of ST@CuONPs against emerging drug-resistant bacteria

#### 2.2.1 Agar disk diffusion method

The antibacterial activity of ST@CuONPs was evaluated against six bacterial strains used in our previous study (Loqman et al., 2022), including *E. faecalis* ATCC 29212 strain, *E. faecium* VREATCC 700221vanA strain, methicillin-susceptible *S. aureus* ATCC 29213 strain, methicillin-resistant *S. aureus* NCTC 12493 strain, susceptible *P. aeruginosa* ATCC 27853 and resistant *P. aeruginosa* ATCC BAA-2108 strain. Antimicrobial activity was assessed by the disk diffusion method (Bauer et al., 1966), and the assays were done in triplicate. Briefly, bacterial strains were cultured on Mueller-Hinton plates and incubated at 30 °C for 18–24 h. Bacterial suspension was then adjusted to 0.5 McFarland turbidity standards, followed by uniform spreading onto Mueller-Hinton agar plates using sterile swabs. Sterile blank paper disks were impregnated with 5, 25, and 45 µL of a 300 μg/mL ST@CuONPs stock solution, corresponding to absolute mass loads of 1.5, 7.5, and 13.5 µg/disk, respectively, and were then placed on top of the agar plates. Commercial antibiotic disks, cefoxitin, ceftazidime and vancomycin (30 µg per disk; bioMérieux, Marcy L’Etoile, France), were used as positive controls. Plates were incubated at 30 °C for 24 h, after which the diameters of the inhibition zones were measured to evaluate antimicrobial activity.

#### 2.2.2 Minimum inhibitory concentrations

Minimum inhibitory concentrations (MICs) of ST@CuONPs against the reference and resistant strains were determined using the broth microdilution method. The experimental setup was adapted from the standard ISO 20776-1:2019 reference methodology regarding the inoculum size, medium (Mueller-Hinton broth), and incubation parameters (ISO, 2019). A 5 × 10^5^ cfu/mL inoculum of each strain was cultured in Mueller-Hinton broth and added to U bottom microtiter plates (Thermo Fisher Scientific, USA) containing ST@CuONPs or gentamycin (a positive control). The plates were incubated for 18–24 h at 30 °C. *Enterococus faecalis* ATCC 29212, methicillin-susceptible *S. aureus* ATCC 29213 and susceptible *P. aeruginosa* ATCC 27853 strains were used as the positive control strain. Iodonitrotetrazolium chloride (INT) was employed as an indicator of microbial growth (Sigma Aldrich, Allemagne), and the assays were done in triplicate.

#### 2.2.3 Removal of preformed biofilms by ST@CuONPs

The crystal violet staining method (Djordjevic et al., 2002) was used to evaluate the effect of different volume of ST@CuONPs (0×, 1/4×, 1/2×, and 1× MIC) on preformed biofilm of six bacterial strains. In a typical assay, overnight bacterial cultures adjusted to 0.5 McFarland were added into Luria-Bertani (LB) broth in a 96-well microplate, in the presence or absence of ST@CuONPs, to a final volume of 200 µL per well. Plates were incubated at 30 °C for 24 h. Following incubation, the supernatants were removed, and the wells were washed with sterile distilled water to remove non-adherent cells. The remaining adherent biofilm was stained with 200 μL of 0.1% crystal violet for 20 min. Finally, 200 μL of 30% (v/v) acetic acid was added to each well to solubilize the bound dye. Biofilm formation was quantified by measuring absorbance at 590 nm using a Chameleon microplate reader (Hidex, Finlande). All experiments were conducted in independent triplicates.

Biofilm inhibition percentage was determined using the following formula:

$$\%\text{ inhibition}=\left(\left(Control-Treated\right)/Control\right)\times 100$$

where control and treated are the bacteria cells in presence and absence of ST@CuONPs.

#### 2.2.4 Statistical analysis

Statistical analysis was performed using Python software (version 3. x). Plotted data were reported as mean ± standard deviation. One-way analysis of variance (ANOVA) followed by a Tukey test was used to detect significant differences in the agar disk diffusion method and the antibiofilm assays.

## 3 Results and discussion

### 3.1 Biosynthesis and analysis method of ST@CuONPs

#### 3.1.1 UV-visible analysis

The kinetic study of the reaction was monitored by UV-Vis spectroscopy over a period of 1–120 h to confirm the formation of ST@CuONPs in aqueous medium. The production of ST@CuONPs was initially assessed by the color change of the reaction mixture from light blue to greenish brown, with the color intensity gradually increasing over the incubation period of up to 120 h. As shown in (Figure 1), the absorption intensity at the 210–220 nm region increased progressively with incubation time. Compared to the baseline profile of the cell-free *S. thinghirensis* supernatant alone, which remains mostly flat and low in this zone (Figure S1), this progressive increase, reflects the continuous complexation of *S. thinghirensis* secondary metabolites during the capping process. Concurrently, the characteristic broad absorption shoulder of the monoclinic CuO between 250 and 300 nm became clearly defined after 24 h and reached its maximum intensity after 120 h. This systematic increase in optical density confirms the steady nucleation and growth of ST@CuONPs. Therefore, an incubation period of 120 h was selected as the optimum reaction time for maximum nanoparticle yield. Similar results have been reported for the green synthesis of CuONPS using *Acalypha indica*, with an optical absorbance around 220 nm (Sivaraj et al., 2014). While, Sathiyavimal et al. (2018) investigated *Sida acuta* leaf extract on the fabrication of CuONPs, their results confirmed biosynthesis of CuONPs at wavelength of 337 nm. It is widely recognized that the SPR bands are very sensitive and highly dependent on nature of the synthesized nanoparticles, size and shape of the particles, the surroundings media, and their inter particle distances (Jain et al., 2007; Raja et al., 2017; Ruttkay-Nedecky et al., 2019).

**FIGURE 1:**
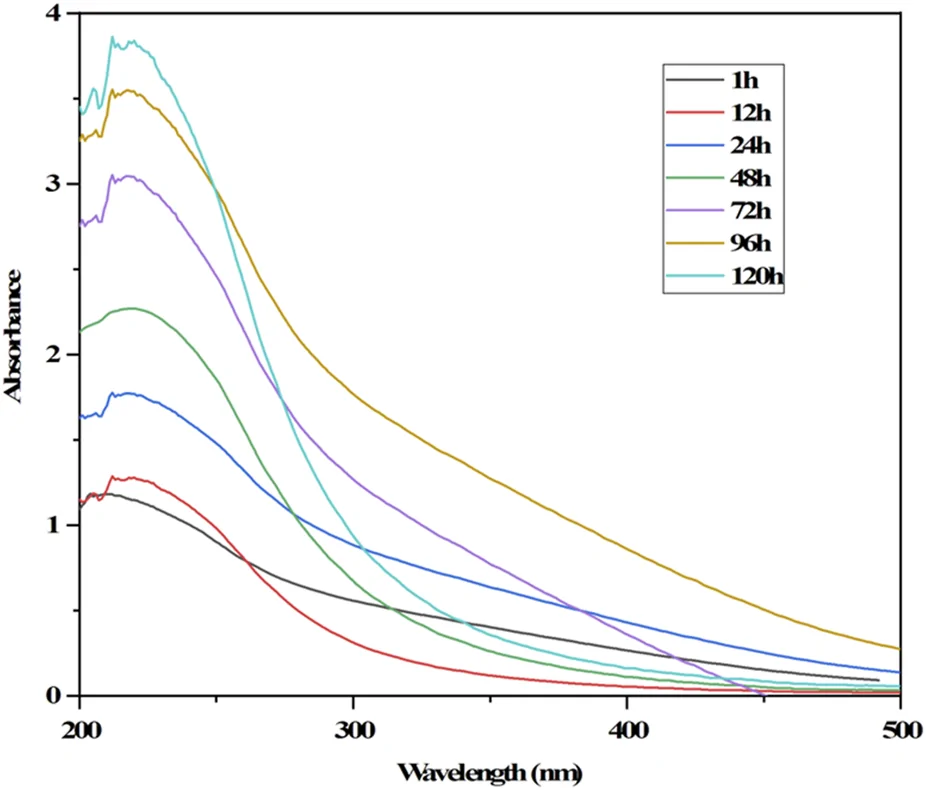
UV–Visible analysis of ST@CuONPs recovered from *S. thinghirensis*.

#### 3.1.2 Dynamic light scattering and zeta potential measurements

The size distribution and surface properties of the synthesized ST@CuONPs were evaluated using Dynamic Light Scattering (DLS) and Zeta potential analysis (Figures 2a,b). The Zeta potential was measured at −13.5 ± 1.8 mV (Figure 2a), indicating that the ST@CuONPs are moderately stable in the dispersion medium. The intensity-weighted DLS trace (Figure 2b) revealed a clearly bimodal size distribution, yielding an overall average hydrodynamic diameter of 54.2 ± 0.3 nm with a polydispersity Index (PDI = 0.30).

**FIGURE 2:**
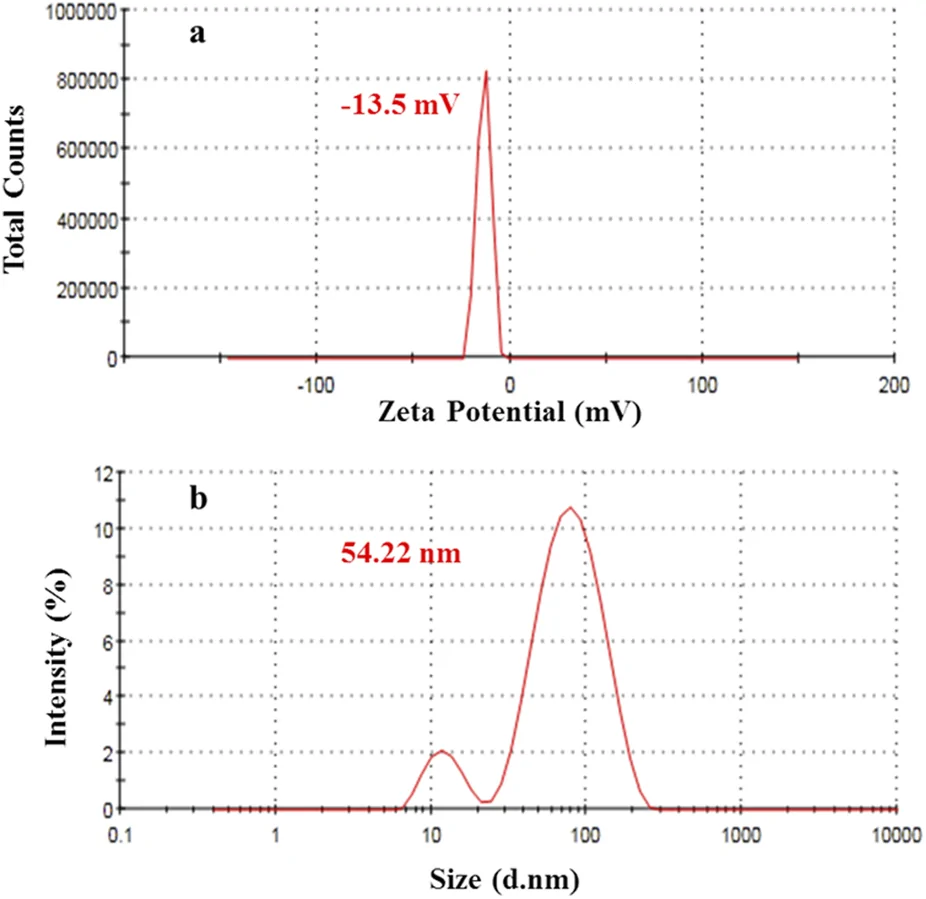
Zetasizer graph of ST@CuONPs: **(a)** Zeta potential and **(b)** particle size.

Our results showed that the ST@CuONPs were moderately stable when compared with those reported by Sankar et al. (2014), which showed that the size and zeta potential distribution of the biosynthesized CuONPs using *Ficus religiosa* were 577 nm and −25.4 mV, respectively. Comparable results were also observed by Nabila et al. using actinomycetes (Nabila and Kannabiran, 2018).

#### 3.1.3 XRD, FTIR, and EDX analysis

The XRD diffraction pattern of ST@CuONPs (Figure 3a) was investigated to determine their crystalline structure and phase purity. The experimental 2-Theta (°) peaks observed at specific positions of 38.04°, 44.16°, 62.05°, 76.80°, and 81.14° correspond respectively to the (111), (200), (−202), (022), and (222) planes of monoclinic CuO. These values are in excellent agreement with the standard JCPDS # 80-0076 and the corresponding COD # 9015924 (Hasan et al., 2022; Siddiqui et al., 2020). The absence of any secondary diffraction peaks of Cu and Cu_2_O phases, confirming the high phase purity of the biosynthesized ST@CuONPs. The experimental and theoretical values of 2θ, and corresponding Miller indices are summarized in Table 1.

**FIGURE 3:**
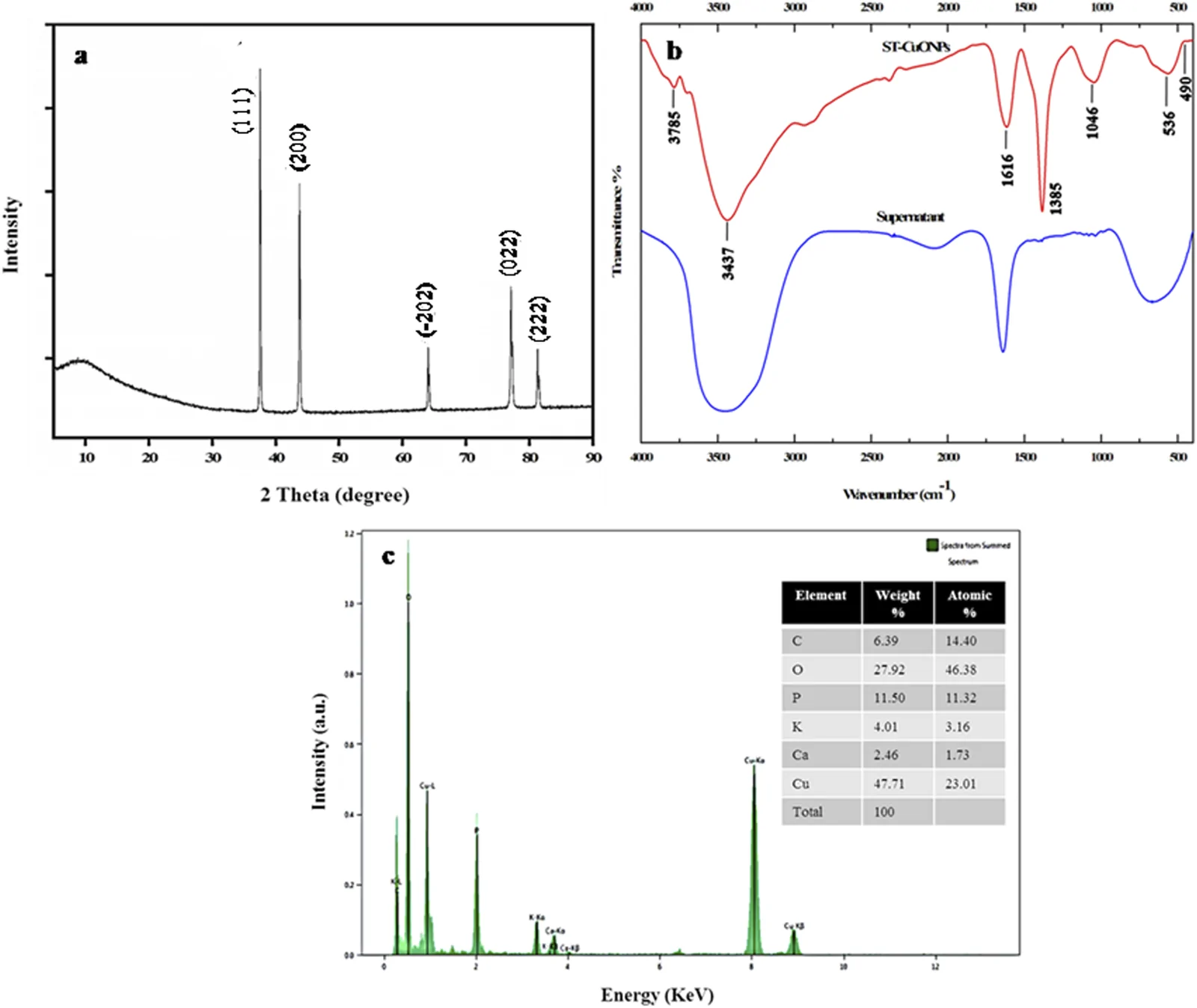
Characterizations of prepared ST@CuONPs: **(a)** XRD pattern, **(b)** FT-IR spectrum of ST@CuONPs compared with supernatant of *S. thinghirensis*, and **(c)** energy dispersive spectrum.

**TABLE 1:** Crystallographic parameters and phase verification for biosynthesized ST@cuONPs: comparison between experimental values and theoretical ICDD data (JCPDS 80-0076) highlighting the calculated standard deviations (Δ 2θ).

Miller indices (hkl)	Experimental 2θ	Theoretical 2θ (JCPDS 80-0076)	Standard deviation (Δ2θ)	Phase
(111)	38.04°	38.12°	0.08°	Monoclinic CuO
(200)	44.16°	44.15°	0.01°	Monoclinic CuO
(-202)	62.05°	62.01°	0.04°	Monoclinic CuO
(022)	76.80°	76.71°	0.09°	Monoclinic CuO
(222)	81.14°	81.25°	0.11°	Monoclinic CuO

Moreover, FTIR analysis was performed to identify the chemical groups present in the synthesized ST@CuONPs, and potentially determine the biomolecules engaged in the reduction mechanism of Cu^2+^ ions by the actinobacteria filtrate. Figure 3b presents a representative FTIR spectrum of the supernatant and ST@CuONPs. The FTIR patterns of supernatant revealed the presence of eminent absorption bonds at ∼1653 (C–O stretching of peptide) and ∼3400 cm^-1^(N–H bending vibration). The spectrum of ST@CuONPs is composed principally of seven characteristic peaks at ∼490, ∼563, ∼1046, ∼1385, ∼1616, ∼3437 and ∼3785 cm^-1^ respectively. The peaks at approximately 490 and 563 cm^−1^ are associated with metal-oxygen (Cu-O) vibrations, confirming the presence of the monoclinic phase in CuONPs (Sivaraj et al., 2014; Rehana et al., 2017). The band at ∼1046 cm^−1^ relates to C–N of amines. The band at ∼1385 cm^−1^ corresponds to N–H bending mode, while that at ∼3437 cm^−1^ is attributed to N–H stretching vibration of primary amine. Finally, the strong broad peak at ∼3785 cm^−1^ relates to the O–H stretching of alcohols and phenols (Nabila and Kannabiran, 2018).

Furthermore, the surface elemental composition of ST@CuONPs showed the predominance of Cu (∼48%) and O (∼28%) (Figure 3c). The others peak of P, C, Ca and K atoms were observed due to X-ray emission from secondary metabolites of *S. thinghirensis*.

#### 3.1.4 TEM analysis

The size and morphology of the synthesized ST@CuONPs were investigated by TEM investigation. The morphological images clearly show well-dispersed, versatile and quasi-spherical shape distribution of ST@CuONPs (Figures 4a–d), with sizes ranging from 6 to 16 nm (∼9.8 ± 0.27 nm) (Figure 4f). The discrepancy between the TEM and the average DLS hydrodynamic size (54.22 ± 0.3 nm) is attributed to the fact that TEM measures the solid inorganic core diameter under dry vacuum conditions, whereas DLS measures the hydrodynamic diameter in a solvated state, including the capping chemical ligands and the surrounding hydration shell. The TEM micrographs also show a certain degree of aggregation of the ST@CuONPs (Figures 4a,b), which can be attributed to the adhesive properties of the actinobacterial secondary metabolites. However, high-magnification images (Figures 4c,d) reveal that the dark copper oxide cores remain separated and encapsulated within a diffuse, lighter organic matrix, acting as a physical barrier that prevents irreversible nanoparticle aggregation. The SAED analysis confirmed the crystallinity of ST@CuONPs with bright circular rings (Figure 4e), where the inter-planar d-spacing were determined to be 2.73, 2.19, 1.91, 1.56 and 1.35 Å, corresponding to the (111), (200), (−202), (022), and (222) lattice planes, respectively. The TEM data agree well in accordance with the standard crystal structure data (JCPDS # 80-0076 and the corresponding COD # 9015924) in DRX analysis. The results show the smaller sizes of the ST@CuONPs when compared with other CuONPs observed in previous reports prepared by other organics extract. Papaya leaf extracts were used to develop CuONPs (Chen et al., 2019). The results revealed that the majority of the CuONPs, approximately 78%, ranged in size from 40 to 80 nm. In another notable study, *Calotropis procera* L. was used to synthesize CuONPs with average size of 15 nm (Harne et al., 2012). It can be concluded from these studies that the types of organic extracts play a crucial role in size variations.

**FIGURE 4:**
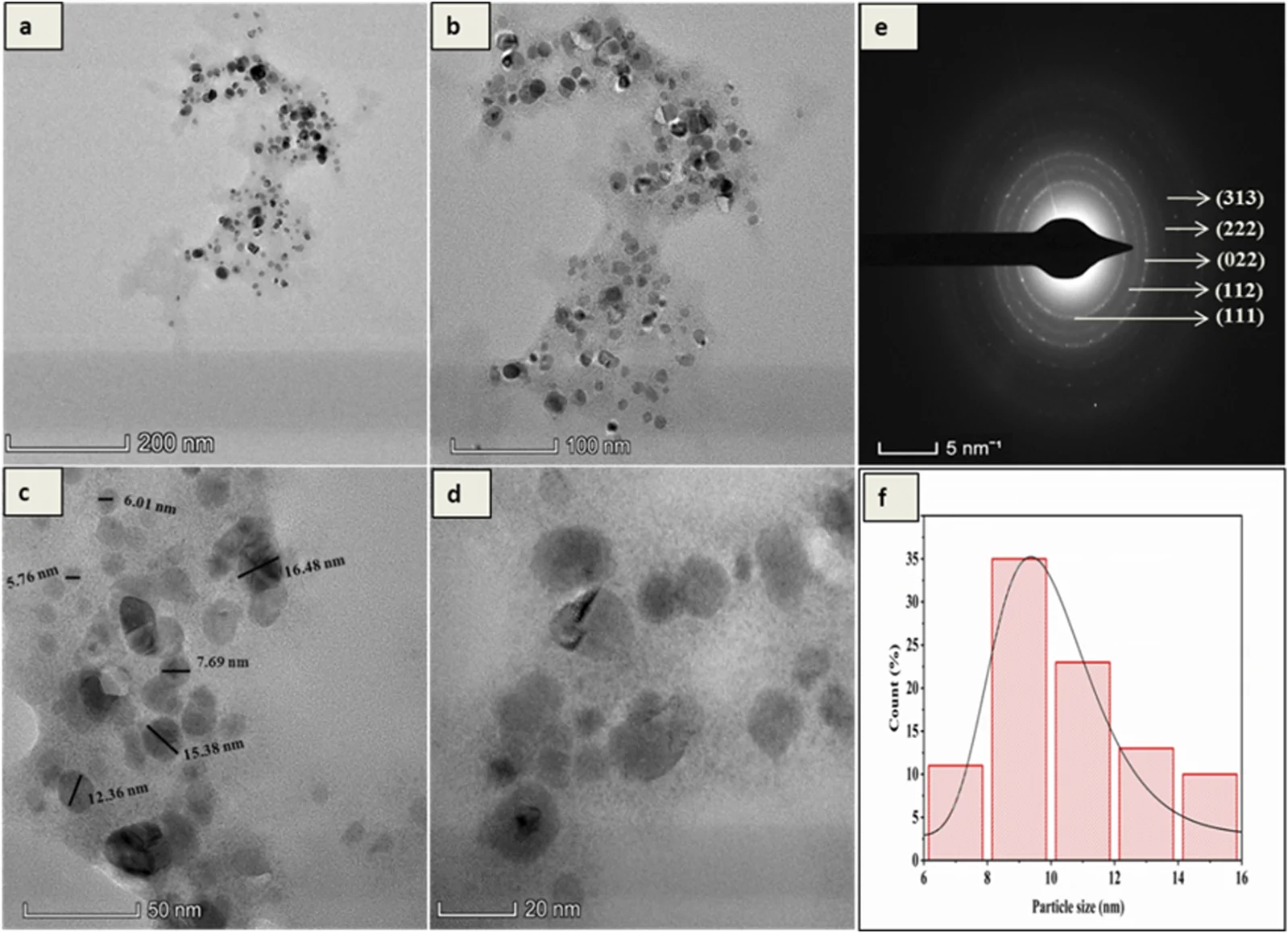
Microscopic study of ST@CuONPs: **(a–d)** TEM analysis, **(e)** SAED pattern and **(f)** particle size distribution curve. SAED: Selected Area Electron Diffraction.

### 3.2 Evaluation of the effect of ST@CuONPs against emerging drug-resistant bacteria

#### 3.2.1 Agar disk diffusion method

The antibacterial potential of ST@CuONPs was evaluated quantitatively by measuring the zone of inhibition against four Gram-positive bacterial strains, including *E. faecalis* ATCC 29212, *E. faecium* VREATCC 700221vanA, *S. aureus* ATCC 29213, Cefoxitin-resistant *S. aureus* NCTC 12493, and two Gram-negative bacterial strains, such as *P. aeruginosa* ATCC 27853 and *P. aeruginosa* ATCC BAA-2108 (Figures 5, 6). The negative control (DMSO) and the lowest dose of ST@CuONPs (1.5 µg/disk) exhibited no inhibitory effect (zone of inhibition = 0.00 mm) against any of the tested pathogens (*p* > 0.05).

**FIGURE 5:**
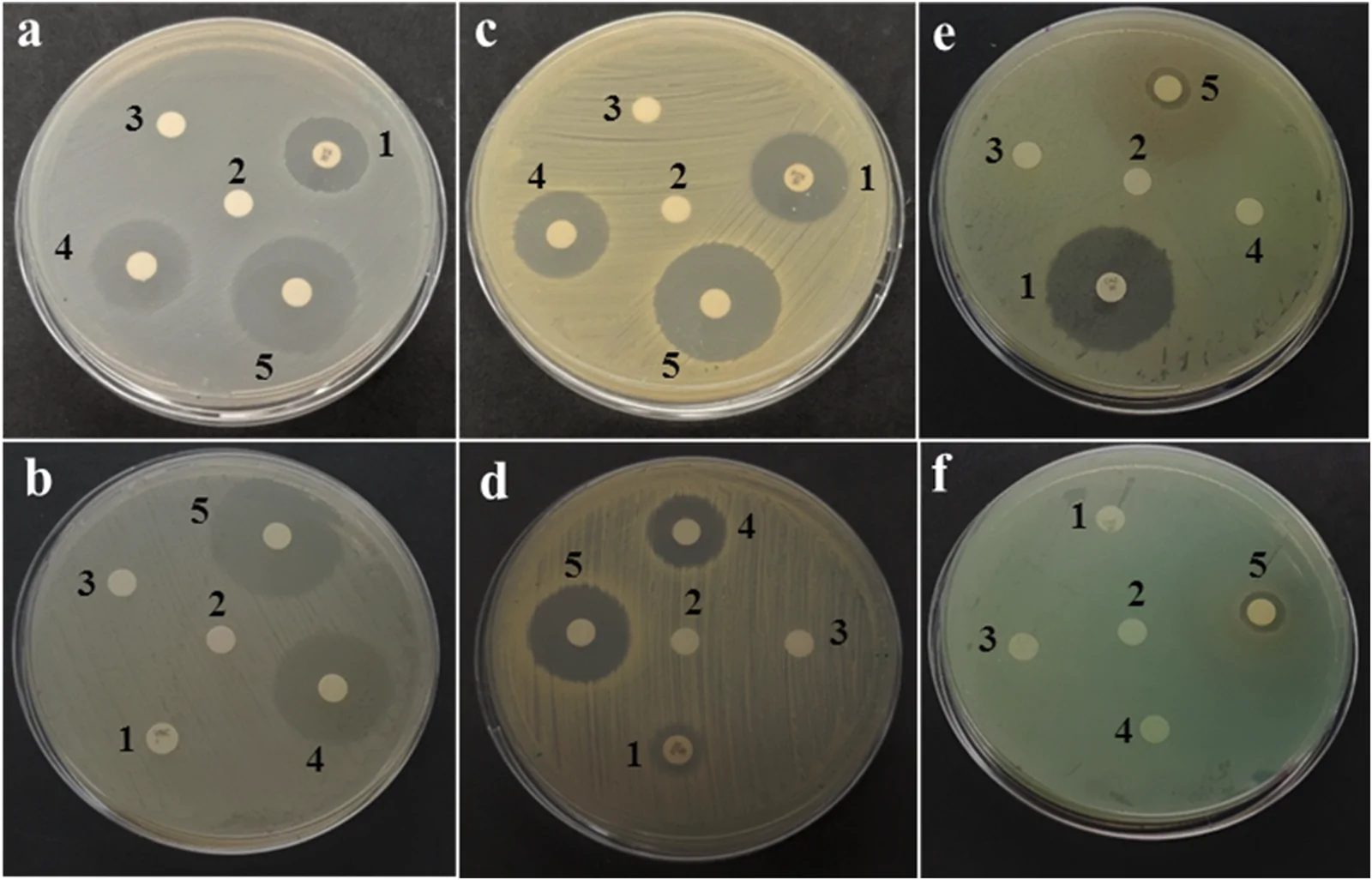
Agar plates showing diameters of zones of inhibition of **(a)** *E. faecalis* ATCC 29212, **(b)** *E. faecium* VREATCC 700221vanA, **(c)** *S. aureus* ATCC 29213, **(d)** *S. aureus* NCTC 12493, **(e)** *P. aeruginosa*ATCC 27853 and **(f)** *P. aeruginosa* ATCC BAA-2108. (Wells 1, 2, 3, 4, and five in the plate were labeled for antibiotics (Vancomycin (* E. faecalis* and *E. faecium*), Cefoxitin(*S. aureus*) and Ceftazidime (*P. aeruginosa*)), DMSO (negative control), 5 µL of ST@CuONPs, 25 µL of ST@CuONPs and 45 µL of ST@CuONPs, respectively).

**FIGURE 6:**
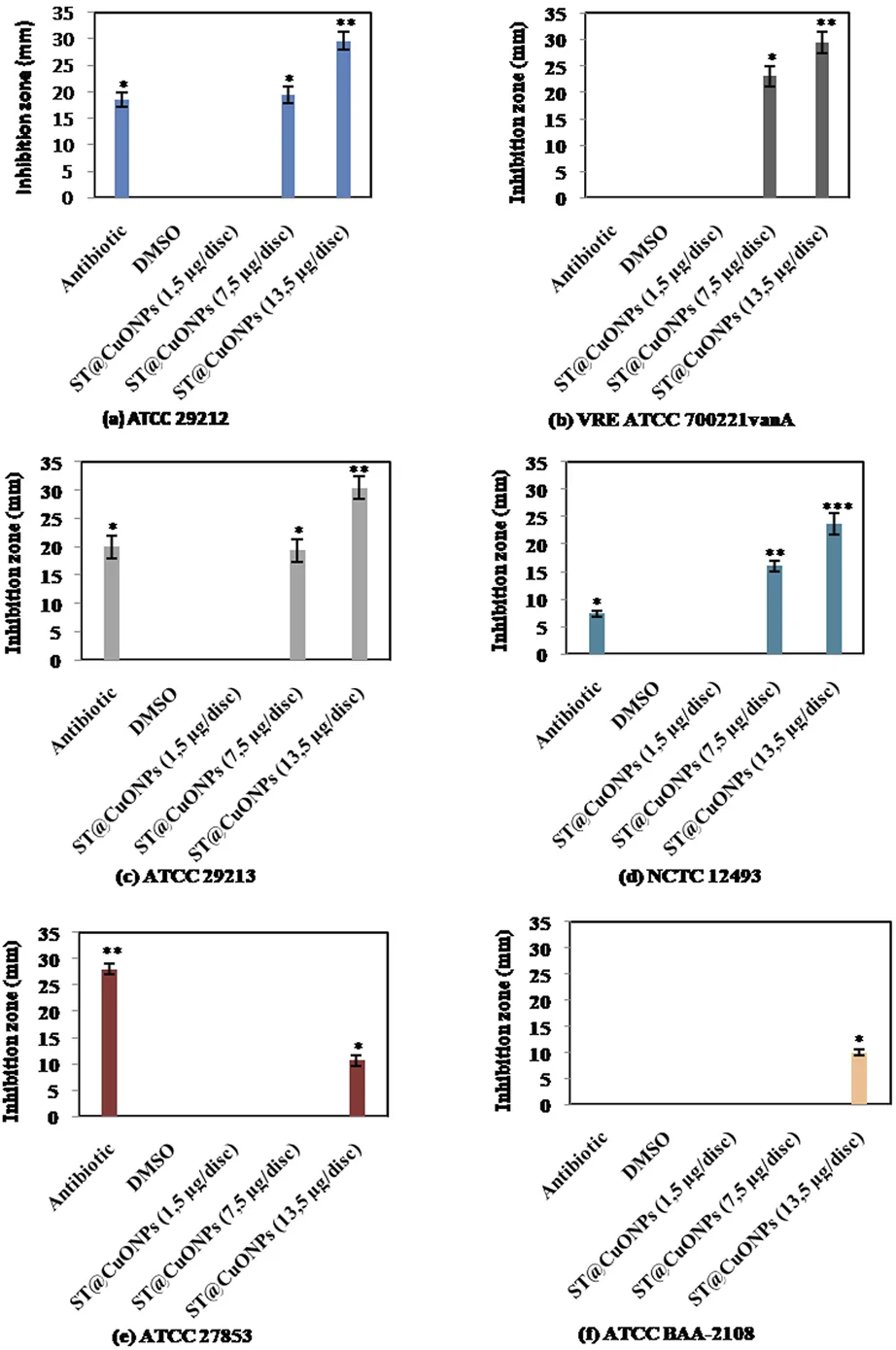
Antimicrobial activity of ST@CuONPs against selected bacteria pathogens: **(a)** *Enterococus faecalis* ATCC 29212, **(b)** *E. faecium* VRE ATCC 700221vanA, **(c)** *S. aureus* ATCC 29213, **(d)** *S. aureus* NCTC 12493, **(e)** *P. aeruginosa* ATCC 27853 and **(f)** *P. aeruginosa* ATCC BAA-2108. Data are presented as mean values ±standard deviation (SD) of independent triplicates (*n* = 3); ***p < 0.001 (extremely significant), **p < 0.01 (highly significant), *p < 0.05 (significant).

Conversely, a prominent, dose dependent antibacterial activity was observed when increasing the ST@CuONPs dose from (7.5 µg/disk) to (13.5 µg/disk). For *E. faecalis* ATCC 29212, *S. aureus* ATCC 29213, and *S. aureus* NCTC 12493, the treatment with (13.5 µg/disk) of ST@CuONPs yielded the significantly highest inhibition zones, reaching 29.00 ± 1.80 mm (Figures 5a, 6a), 30.00 ± 2.00 mm (Figures 5c, 6c), and 23.00 ± 2.00 mm (Figures 5d, 6d), respectively. These values were statistically superior to those obtained with both the (1.5 µg/disk) treatment and the standard antibiotic (*p* < 0.05).

Remarkably, ST@CuONPs demonstrated strong efficacy against highly resistant strains. The *E. faecium* VRE ATCC 700221vanA and *P. aeruginosa* ATCC BAA-2108, which were completely resistant to the reference antibiotic (zone of inhibition = 0.00 mm), showed high susceptibility to ST@CuONPs. Specifically, (13.5 µg/disk) of ST@CuONPs produced a substantial inhibition zone of 29.17 ± 2.02 mm against *E. faecium* VRE ATCC 700221vanA (Figures 5b, 6b) and 10.00 ± 2.00 mm against *P. aeruginosa* ATCCBAA-2108 (Figures 5f, 6f) (*p* < 0.05). This noteworthy finding highlights the potential of ST@CuONPs as potent alternative agents to overcome conventional antibiotic resistance mechanisms.

In contrast, for *P. aeruginosa* ATCC 27853 (Figures 5e, 6e), the positive control antibiotic (Ceftazidime) remained the most effective agent (28.00 ± 1.00 mm), although a significant inhibitory effect was still initiated at (13.5 µg/disk) of ST@CuONPs (10.00 ± 1.00 mm). These statistical analyses confirm that the synthesized ST@CuONPs possess robust and broad-spectrum antibacterial properties.

A limitation of the current study is the absence of a concurrent, internal cell-free supernatant control performed within the exact same experimental run. Nevertheless, given that our previous work established only a minor baseline effect for the supernatant alone (12˗14 mm) (Loqman et al., 2022), the remarkably larger zones of inhibition achieved here strongly indicate that the enhanced antibacterial potency is driven by the newly synthesized ST@CuONPs.

Many studies are in line with our work. It has been observed that nanoparticles exhibit stronger activity against Gram-positive bacteria compared to Gram-negative bacteria (Wang et al., 2017), while others revealed contrary results (Roy et al., 2019). Priya et al. (2023) fabricated CuONPs utilizing *Morinda citrifolia* leaves and tested their potential against three bacteria (*Escherichia coli*, *B. subtilis*, and *S. aureus*). The main results demonstrated that CuONPs exhibit greater effectiveness against *B. subtilis* compared to the other two bacteria (*E. coli* and *S. aureus*). It achieved a 13.0 mm zone of inhibition at a concentration of 25 µL.

#### 3.2.2 Minimum inhibitory concentrations

The broth microdilution assay is the most suitable method for determining MIC values. This susceptibility testing method was employed to assess the antimicrobial effect of various concentrations of ST@CuONPs varying from 3.152 to 100 μg/mL (Figure 7). The results of the antibacterial assay indicated that ST@CuONPs demonstrated a stronger bacterial inhibitory effect against the Gram-positive bacterial strains compared to the Gram-negative strains.

**FIGURE 7:**
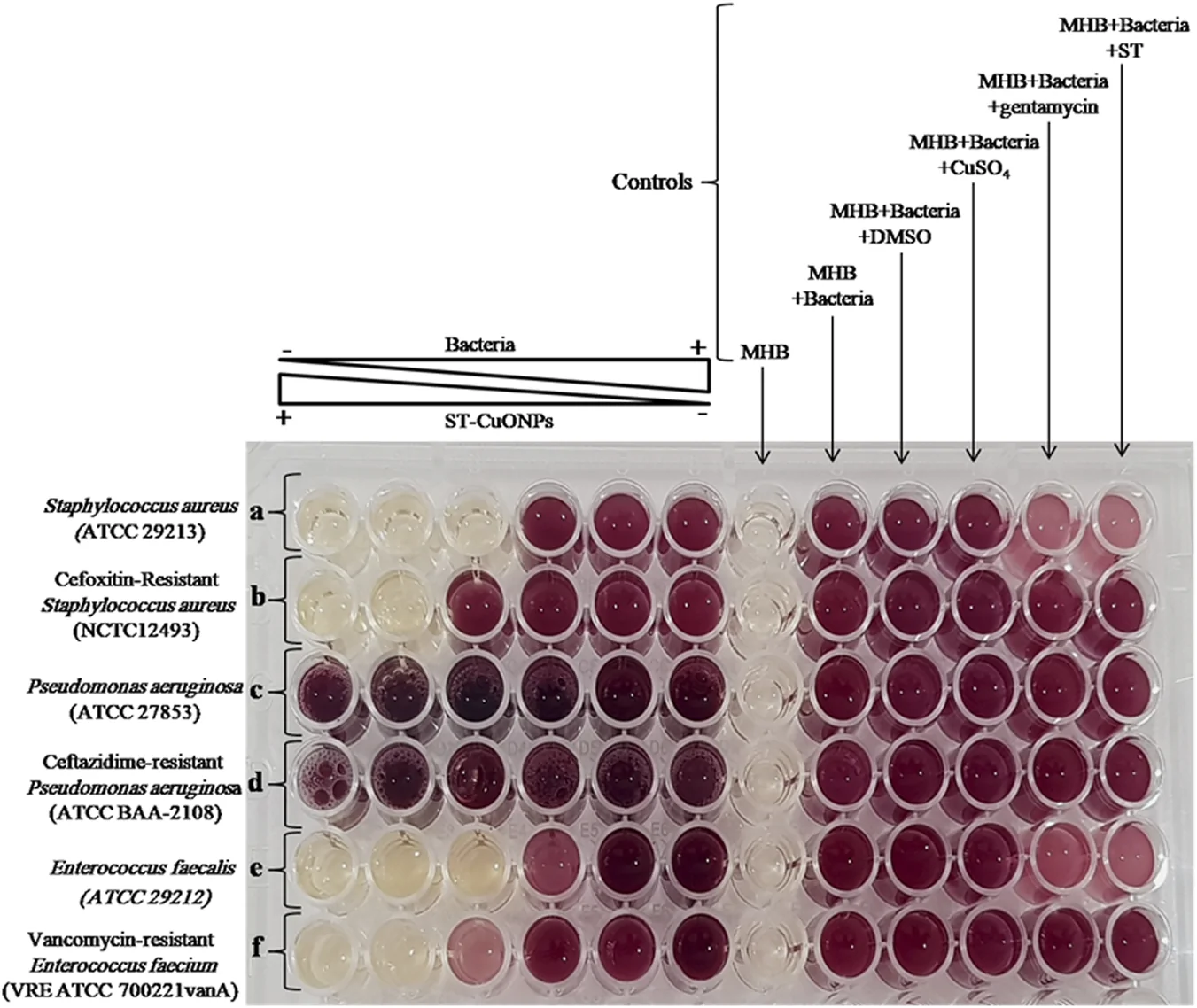
Microdilution assay results of ST@CuONPs against **(a)** *E. faecalis* ATCC 29212, **(b)** *E. faecium* VRE ATCC 700221vanA, **(c)** *S. aureus* ATCC 29213, **(d)** *S. aureus* NCTC 12493, **(e)** *P. aeruginosa* ATCC 27853 and **(f)** *P. aeruginosa* ATCC BAA-2108.

Table 2 and Figure 7 indicate the result of microdilution method. The ST@CuONPs showed significant activity against native bacteria. *Enterococus faecalis* ATCC 29212 and *Staphylococus aureus* ATCC 29213 have the same MIC (12.5 μg/mL). Conversely, ST@CuONPs displayed limited inhibitory activity against *P. aeruginosa* ATCC 27853. Regarding MDR strains, the antimicrobial activity against *E. faecium* VRE ATCC 700221vanA was similar to that of *S. aureus* NCTC 12493, with an MIC value of 25 μg/mL. However, ST@CuONPs showed less activity against *P. aeruginosa* ATCC BAA-2108. Our results are consistent with other studies, where they agreed that the efficiencies of the nanoparticles were each observed to be dependent on the concentration, which means that the efficiency increased with increasing the nanoparticles concentration used for bacteria treatment. The doses used in this study are lower than those previously reported in other research. Ahamed et al. (2014) demonstrated that CuONPs possess antibacterial activity against *E. coli* and *E. faecalis*, with MIC values of 31.25 μg/mL, while *K. pneumoniae* exhibited a MIC of 250 μg/mL. Another significant study on CuONPs synthesized from *Athrixia phylicoides* DC demonstrated that the most effective antimicrobial activity was observed at concentrations of 0.62 mg/mL (*Bacillus cereus*) and 0.16 mg/mL (*S. aureus*) (Kaningini et al., 2023).

**TABLE 2:** Values of minimal inhibitory concentration of ST@CuONPs against selected bacteria.

Gram status	Bacterial strains tested	MIC (µg/mL)
Gram-positive	*E. faecalis* ATCC 29212	12.5
	*E. faecium* VREATCC 700221vanA	25
	*S. aureus* ATCC 29213	12.5
	*S. aureus* NCTC 12493	25
Gram-negative	*P. aeruginosa* ATCC 27853	100
	*P. aeruginosa* ATCC BAA-2108	100

#### 3.2.3 Antibacterial mechanisms of ST@CuONPs

CuONPs are widely used in biomedicine as antimicrobial agents (Arakawa et al., 2020; Erci et al., 2020; Sharif et al., 2021). Only a few studies have explored the detailed antibacterial mechanisms of CuONPs. Researchers investigated this nanomaterial for its potential as a novel antimicrobial agent due to its small size and its large intake by bacteria (Yoon et al., 2007; Usman et al., 2013; Hassan et al., 2019). The key action points of CuONPs are the bacterial wall, the ribosome, the genetic materials and the plasma membrane.

Based on comprehensive insights from prior literature, we propose a hypothetical framework to describe the potential antibacterial mechanism of our biosynthesized ST@CuONPs (Figure 8; Table 3). The bactericidal effect of CuONPs is directly related to one of four mechanisms, such as generation of reactive oxygen species (ROS), impairment of bacterial cell membranes, DNA damage and inhibition of protein synthesis (Zakharova et al., 2015; Dakal et al., 2016). Generally, CuONPs adhere to the microbial cell surface and penetrate bacterial cells by interacting with the cell membrane, facilitating the passage of Cu^2+^ ions. Once inside, CuONPs disrupt cellular organelles and biomolecules, interfering with the electron transport chain and bacterial metabolic regulation (Ma et al., 2022; Devaraji et al., 2024). The antibacterial activity of CuONPs is influenced by their physicochemical properties as well as the thickness and composition of the bacterial cell wall. Numerous studies have demonstrated that nanoparticles exhibit stronger activity against Gram-positive bacteria than Gram-negative bacteria (Wang et al., 2019), likely due to structural differences in their cell walls. In Gram-positive bacteria, peptidoglycan is the primary component of the cell membrane, whereas in Gram-negative bacteria, approximately 90% of the cell wall is composed of lipids. Since copper has a higher affinity for proteins than for lipids, the thicker peptidoglycan layer in Gram-positive bacteria (around 30 nm) is more susceptible to CuONPs interaction compared to the thinner layer in Gram-negative bacteria (approximately 3˗4 nm) (Dakal et al., 2016).

**FIGURE 8:**
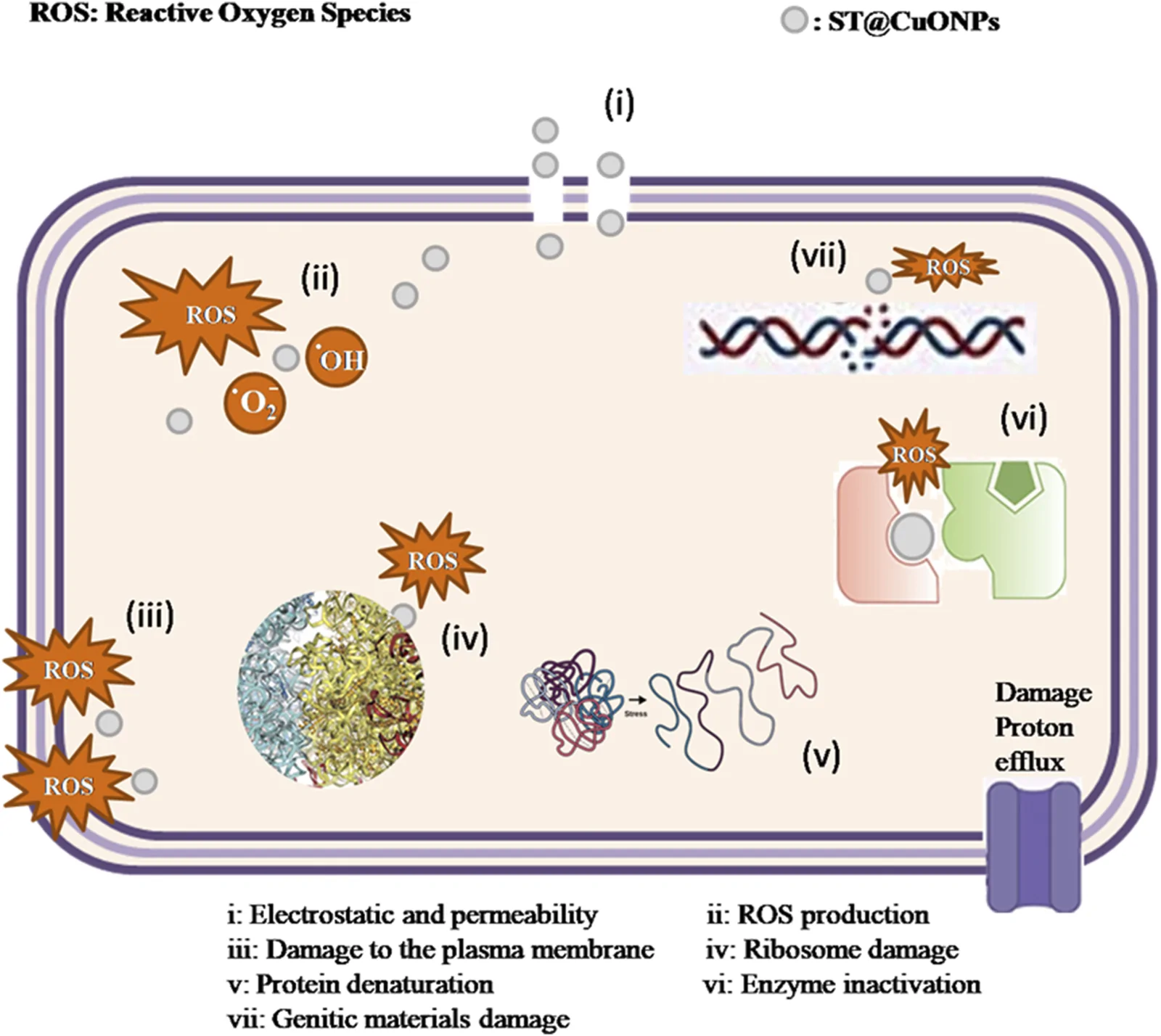
Schematic representation of the proposed antibacterial mechanism of biosynthesized ST@CuONPs.

**TABLE 3:** Mechanisms of antibacterial effect of CuONPs.

Bacterial strain	CuONPs Size (nm)	Mode of action	Method	References
*P. aeruginosa *	25	Interaction with cell membrane; interaction with the main components of biomolecules in the bacterial cell (S and P)	TEM and STEM-HAADF	(España-Sánchez et al., 2017)
*E. coli * *S. aureus*	20–95	disruptcell membrane and cellular function	-	(Ren et al., 2009)
*E. coli * *S. aureus *	5	Cell membrane damage; Electrostatic interaction between Cu ions and membrane anionic phospholipids	Atomic force microscope (AFM)	(Bogdanović et al., 2014)
*S. aureus* *B. cereus* *P. aeruginosa* *E. coli*	5–22	production of ROS and membrane disruption	Scanning electron microscopy (SEM)	(Gopinath et al., 2016)
*Aggregatibacter actinomycetemcomitans* *Porphyromonas gingivalis* *Prevotella intermedia* *Fusobacterium nucleatum*	-	disruption of cellular function generation of ROS	-	(Vargas-Reus et al., 2012)
*E. coli S. aureus E. faecalis *	5.67	DNA degradation	-	(DeAlba-Montero et al., 2017)
*E. coli*	-	protein oxidation generation of ROS DNA degradation lipid peroxidation	-	(Chatterjee et al., 2014)
*Bacillus subtilis* *Salmonella typhimurium* *P. aeruginosa* *E. coli* *Streptococcus pneumonia* *Proteus mirabilis* *S. aureus* *Staphylococcus saprophyticus*	10–30	accumulation of metal nanoparticles generation of reactive oxygen species (ROS)	-	(Hemmati et al., 2020)
*S. aureus* *E. coli*	18	protein oxidation formation of reactive oxygen species DNA damage	-	(Jadhav et al., 2017)

#### 3.2.4 Removal of preformed biofilms by ST@CuONPs

The effect of different concentrations (0 MIC, 1/4 MIC, 1/2 MIC and 1 MIC) of ST@CuONPs on the biofilm eradication of six bacterial strains is presented in Figure 9. The statistical analysis *via* One-way ANOVA followed by Tukey’s *post hoc* test indicated that the reduction was significant (p < 0.05) and dependent on ST@CuONPs concentrations. In the absence of nanoparticles (0 MIC), all strains exhibited robust biofilm architectures. The highest inhibition of biofilm was observed in Gram-positive bacterial strains while the least inhibition was observed in Gram-negative bacteria. For instance, the strain *Enterococcus faecalis* ATCC 29212 (Figure 9a) exhibited the highest sensitivity, with only 24% ± 2.00% of biofilm remaining at 1/4 MIC (representing a 76% inhibition), which further reduced to 8% ± 2.08% at 1 MIC (92% eradication). A similarly prominent antibiofilm activity was observed for *E. faecalis* VRE ATCC 700221vanA (Figure 9b), which decreased by 64% ± 0.58%, 83% ± 3% and 89% ± 1.15% at 1/4, 1/2 and 1 of MIC of ST@CuONPs, respectively. Regarding the activity against *S. aureus* ATCC 29213 (Figure 9c), the inhibition percentage of biofilm was measured at 68% ± 3.06%, 75% ± 1.15% and 87% ± 1.53% for 1/4, 1/2 and 1xMIC of ST@CuONPs, respectively. However, the treatment of the *S. aureus* NCTC 12493 (Figure 9d) biofilms with ST@CuONPs reduced the amount of stained biofilms to 63% ± 2.52% (1/4×MIC), 72% ± 2.5% (1/2 × MIC) and 86% ± 0.58% (1 × MIC) of pretreatment levels in 24 h. In the comparison of Gram-negative bacteria and untreated bacterial biofilms, ST@CuONPs exhibited significant inhibitory activity; staining and quantification of *P. aeruginosa* ATCC 27853 (Figure 9e) and *P. aeruginosa* ATCC BAA-2108 (Figure 9f) biofilms showed that the 1 × MIC of ST@CuONPs had a modest effect, 68% ± 0.58% and 58% ± 2.52%, respectively. Our findings are consistent with those of previous studies, where CuONPs synthesized using eco-friendly technology, and which were used to eradicate the antibacterial and antibiofilm activity. In a recent report by Shehabeldine et al. (2023) CuONPs synthesized from the live cell filtrate of the fungus *Penicillium chrysogenum* have excellent antibiofilm activity against *K. oxytoca* (49%) and *E. coli* (59%), Whereas an opposite effect was observed against *B. cereus* and *S. aureus*.

**FIGURE 9:**
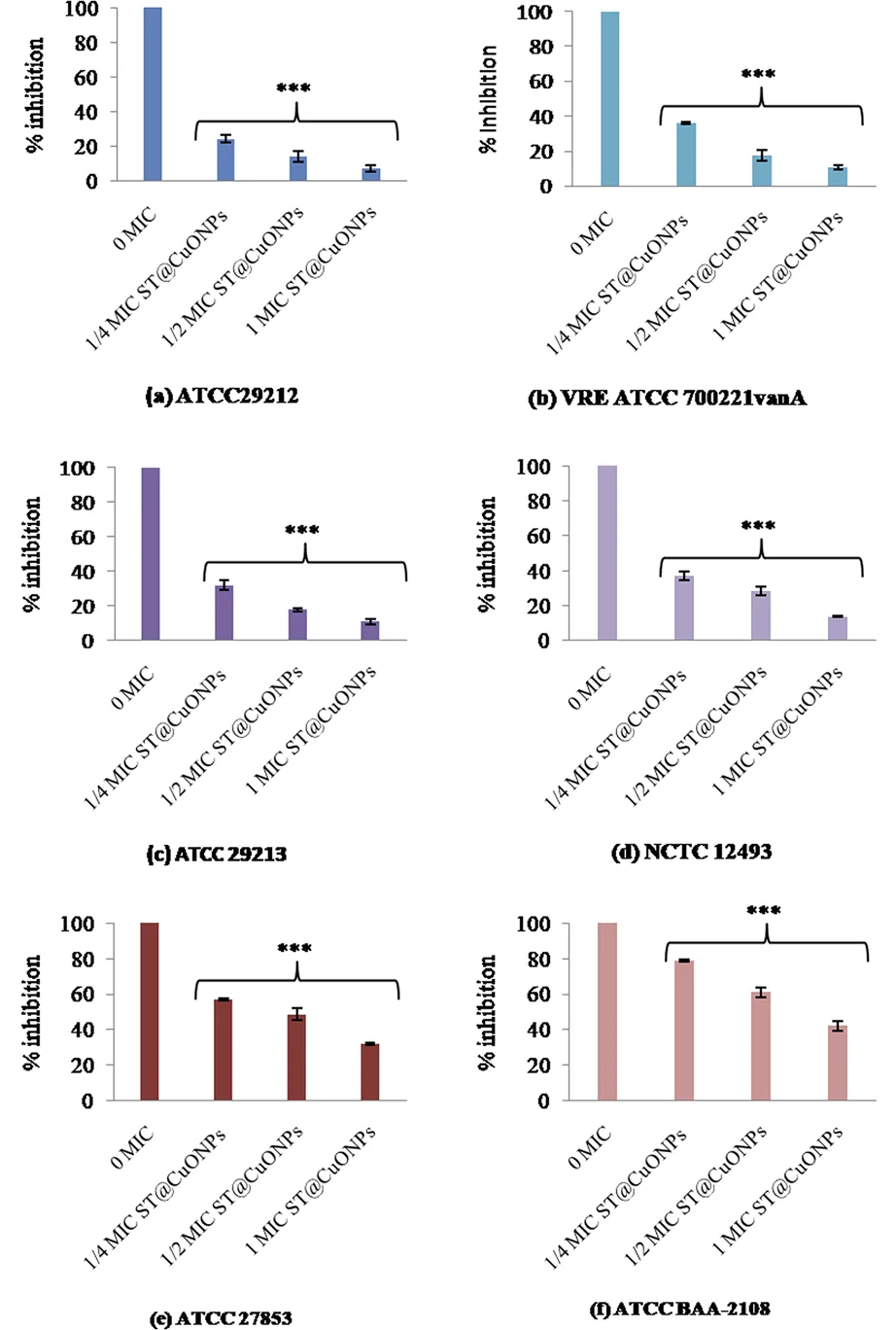
The effect of ST@CuONPs on the biofilm eradication against **(a)** *E. faecalis* ATCC 29212, **(b)** *E. faecium* VRE ATCC 700221vanA, **(c)** *S. aureus* ATCC 29213, **(d)** *S. aureus* NCTC 12493, **(e)** *P. aeruginosa* ATCC 27853 and **(f)** *P. aeruginosa* ATCC BAA-2108. Data are presented as mean values ±standard deviation (SD) of independent triplicates (*n* = 3); ***p < 0.001 (extremely significant).

## 4 Conclusion

In this study, ST@CuONPs were successfully synthesized using an eco-friendly approach based on *S. thinghirensis* metabolites, yielding spherical nanoparticles. The biosynthesized nanoparticles exhibited broad-spectrum antibacterial and antibiofilm activities, with greater efficacy against Gram-positive than Gram-negative MDR strains. These findings highlight the potential of biologically synthesized CuONPs as promising alternative antimicrobial agents. Overall, this green synthesis strategy represents a sustainable and effective approach for developing novel biomaterials to combat antimicrobial resistance.

## Data Availability

The datasets presented in this study can be found in online repositories. The names of the repository/repositories and accession number(s) can be found in the article/Supplementary Material.
